# Effect of Fixatives and Fixation Period on Morphology and Immunohistochemistry of Feline Ovarian Tissue

**DOI:** 10.3390/ani14060825

**Published:** 2024-03-07

**Authors:** Isa Mohammed Alkali, Martina Colombo, Olga Rodak, Wojciech Nizanski, Gaia Cecilia Luvoni

**Affiliations:** 1 Department of Veterinary Medicine and Animal Sciences, University of Milan, 26900 Lodi, Italy; isa.mohammed@unimi.it (I.M.A.); cecilia.luvoni@unimi.it (G.C.L.); 2 Department of Theriogenology, University of Maiduguri, Maiduguri 600230, Nigeria; 3 Department of Histology and Embryology, Wroclaw Medical University, 50-368 Wroclaw, Poland; olga.rodak@student.umw.edu.pl; 4 Department of Reproduction and Clinic for Farm Animals, Wroclaw University of Environmental and Life Sciences, 50-366 Wroclaw, Poland; wojciech.nizanski@upwr.edu.pl

**Keywords:** histology, form acetic, Bouin solution, neutral buffered formalin, DAB intensity, follicle grading

## Abstract

**Simple Summary:**

Reliable assessment of ovarian tissue depends on the ability of the fixation protocol to preserve its near in vivo appearance and composition. The goal of this study was to find the most suitable fixation protocol for feline ovarian tissue. Equal ovarian tissue fragments were obtained and divided into three groups then fixed with three different fixatives (Bouin, neutral buffered formalin [NBF] and form acetic acid) for five time points and assessed for histology and immunohistochemistry. Bouin exhibited good results for morphology but not immunohistochemistry, while NBF exhibited the best results for immunohistochemistry but not morphology. The most suitable fixative for both morphology and immunohistochemistry was form acetic acid, which invariably makes it a better alternative for feline ovarian tissue studies.

**Abstract:**

Fixatives and fixation protocol have a profound effect on both the morphology and epitope sensitivity of ovarian tissue, which hampers accurate ovarian tissue evaluation. We aimed to establish the most suitable fixation protocol for feline (*Felis catus*) ovarian tissue. Fragments (1.5 mm diameter) were punched from 1 mm-thick feline ovarian tissue, divided into three groups then fixed with three different fixatives (Bouin, neutral buffered formalin [NBF] and form acetic acid [new compound fixative formulation for ovarian tissue composed of 5% acetic acid in NBF]) for five fixation periods. Subsequently, fragments were processed and evaluated for the morphology and intensity of immunohistochemical signals against three antigens (Ki-67, MCM-7 and activated caspase-3). Proportions of grade 1 or morphologically intact follicles were significantly lower in NBF when compared with Bouin and form acetic acid fixatives. However, Bouin fixative had the lowest mean DAB intensity (*p* < 0.05) in all three antigen targets, while NBF had the highest (*p* < 0.05) in Ki-67 and caspase-3, but in MCM-7, it was no different from form acetic acid. In conclusion, form acetic acid maintained ovarian tissue architecture with excellent follicular morphology in the same manner as Bouin fixative, and it also maintained reasonable DAB signals similar to NBF, thus providing a better alternative for feline ovarian tissue studies.

## 1. Introduction

Ovarian tissue preservation is an emerging technique of fertility preservation that is gaining recognition in the scientific community, perhaps due to its versatility [1]. It can be carried out in any individual without any hindrance of age or reproductive cycle. This is especially essential in cancer patients, who are at higher risk of losing their fertility due to gonadotoxic therapies [2]. Similarly, this technique fits well with the strategies of conservation of biodiversity or genetic salvage, wherein precious animals have to undergo gonadectomy or euthanasia or are otherwise found dead [3,4]. This is especially essential in endangered families, notably the wild Felids, where there is an absolute need to preserve the little genetic resource whenever there is a chance to do so. Thus, the relative adequacy of the domestic cat population and the availability of their ovaries provide a viable option for refining studies for the future translation to the entire Felidae, especially considering both the structural and ultrastructural similarities of their ovaries [5,6]. One of the limitations of this technique is the lack of unified ovarian tissue evaluation parameters resulting from multiple tissue processing techniques and the different tissue fixatives used, notwithstanding some species variation in ovarian tissue architecture [7].

Fixatives and fixation protocols have a profound effect on both morphology and epitope sensitivity to antibodies during immunostaining, and on even nucleic acid detection [8,9,10]. Fixation is a process whereby tissue is preserved in its near-live macro and microscopic morphology, in addition to preservation of tissue antigen that enables immunorecognition [11]. Therefore, optimum fixation is the foundation for the subsequent assessment of tissue integrity, ranging from basic histology to complex molecular procedures such as immunohistochemistry, in situ hybridization and polymerase chain reaction (PCR) [12].

Several fixatives have been in use since the development of histology in the last century, with the most common being formaldehyde-based fixatives [13]. Although there is no perfect fixative [14], neutral buffered formalin (NBF) has become the most widely used globally for the fixation of soft tissues due to its versatility in fixing a wide range of tissues [15]. However, tissue fixation with NBF has been associated with morphological alteration and artifacts, including shrinkage [16]. Although this is a general trait of formaldehyde-based fixatives, Bouin solution has been reported to be a better option for preservation of tissue morphology [17]. On the other hand, NBF is regarded as superior in terms of the maintenance of tissue antigenicity for immunostaining [18]. The common practice is to place tissues in sufficient NBF and keep them overnight or for up to 24 h; however, a prolonged fixation period leads to the loss of antigenicity due to the excessive crosslinking of protein segments [7,9].

Several factors, including the type of tissue and the dimension and temperature of the fixative, are proportional to the rate of tissue penetration and the duration required for optimum fixation [7,15]. In addition, very little is known of the interactions between fixatives and ovarian tissue fragments and their effects on morphology and immunohistochemistry, especially considering the emerging techniques of ovarian tissue preservation, wherein small ovarian cortical fragments are used. We therefore aimed to find the most suitable fixative for feline ovarian tissue by comparing three different fixatives (Bouin’s solution, NBF and a hybrid compound fixative called form acetic acid [NBF + 5% acetic acid]) for feline ovarian tissue at five different periods each (1, 4, 8, 12 and 24 h).

## 2. Materials and Methods

### 2.1. Reagents and Chemicals

Reagents and chemicals used herein were procured from Sigma–Aldrich (Taufkirchen, Germany) unless otherwise specified.

### 2.2. Ovary Transport and Preparation of Ovarian Fragments

Feline (*Felis catus*) ovaries were obtained as byproducts of elective ovariectomy from veterinary clinics and/or animal shelters then immediately transported to the laboratory in a solution of normal saline and antibiotic [AB: penicillin and streptomycin 5 U/mL (Thermofisher Scientific, Waltham, MA, USA) in Dulbecco phosphate-buffered saline (DPBS)]. Only ovaries with a uniform bean shape were used; thus, ovaries with large corpora lutea were avoided. Ethics committee approval was not required because ovaries were collected during routine ovariectomy. Ovaries from eleven cats were used for the six biological replicates of the study. A total of 15 ovarian cortical tissue fragments were punched from each ovary using a 1.5 mm-diameter biopsy punch (Kai medical, Oyana, Japan) from a 1 mm-thick feline ovarian tissue cortex slice [19].

### 2.3. Experimental Design

**Figure 1:**
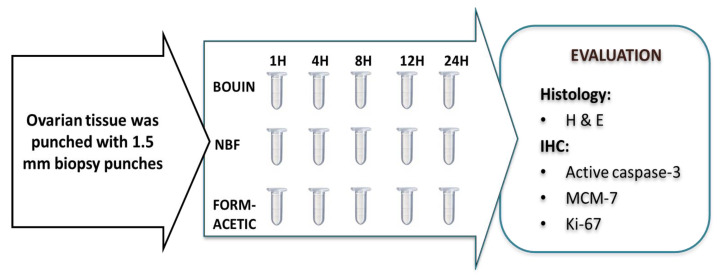
Experimental flow chart of feline ovarian tissue fixation and evaluation. NBF = neutral buffered formalin; H & E = hematoxylin and eosin; IHC = immunohistochemistry; MCM-7 = minichromosome maintenance protein complex component-7 (a proliferation marker associated with the initiation and elongation of DNA replication); Ki-67 = conventional intranuclear proliferation marker with high expression in mitotic cells.

The experimental design has been simplified with a flow chart (Figure 1). Briefly, equal ovarian tissue fragments were divided into three groups and fixed with three different fixatives viz. Bouin, NBF and form acetic acid (a new compound fixative formulation for ovarian tissue composed of 5% acetic acid in NBF) [20]. Each group had five sub-groups based on five durations of fixation (1 h, 4 h, 8 h, 12 h and 24 h). Finally, the fragments were processed and evaluated for the morphology and intensity of immunohistochemical signals.

### 2.4. Histology

Routine histology, including dehydration, paraffin impregnation, embedding and sectioning, was performed manually [21]. Briefly, ovarian fragments were placed in biopsy cassettes (Bio-Optica, Milan, Italy) and submerged in ascending concentrations (two steps for each concentration) of ethanol (70%, 90% and 100%) for 15 min each. The fragments were then exposed to two steps of xylene for 15 min each before being submerged in two-step liquified paraffin for 1 h each and embedded afterwards. Blocks were sectioned (5 µm thickness), and every sixth section was placed on a glass slide and dried overnight on a slide dryer at 37 °C. Slides were stained with hematoxylin and eosin, cover slipped with a xylene-based mounting media (Bio-Optica, Milan, Italy) and digitally scanned with NanoZoomer S60 (Hamamatsu, Shizuoka, Japan).

#### Image Analysis (Hematoxylin and Eosin)

**Figure 2:**
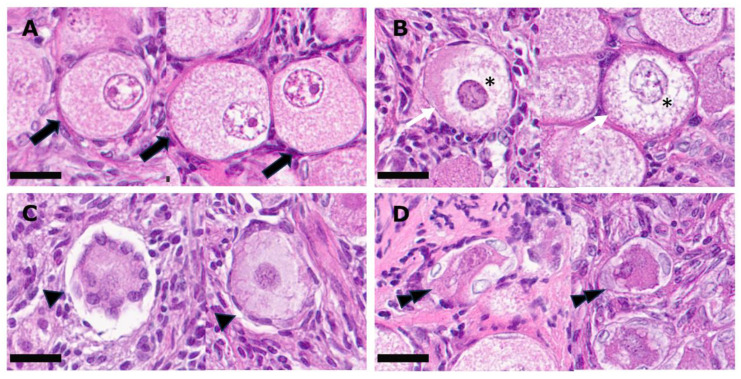
Hematoxylin- and eosin-stained sections of feline ovarian tissue showing different follicle gradings: (**A**) grade 1 (block arrow: spherical in shape, evenly distributed follicular cells, intact stroma, spherical oocyte, intact nucleus and nucleolus and homogenous cytoplasm); (**B**) grade 2 (white line arrow: spherical in shape, evenly distributed follicular cells, intact stroma and spherical oocyte, misshapen nucleus and/or not homogenous cytoplasm [*]); (**C**) grade 3 (arrow head: follicular cells pulled away from the stroma but oocyte spherical); (**D**) grade 4 (double arrow heads: follicular cells pulled away from the stroma and oocyte misshapen, vacuolated, pyknotic nucleus and/or disorganized granulosa cells. Scale bar = 25 µm.

Scanned slides were evaluated using viewer software NDP.view.2.9.29 (Hamamatsu, Shizuoka, Japan). Follicles were counted and graded into four grades (Figure 2) and further classified based on developmental stages (Table 1). Follicles were expressed as a percentage of the total follicles counted, and only those with visible nuclei were counted.

### 2.5. Immunohistochemistry

Immunohistochemical staining was performed automatically [22] in a DAKO Autostainer (Dako, Santa Clara, CA, USA) following the manufacturer’s instructions for the three proteins assessed, namely: Ki-67, MCM-7 (minichromosome maintenance protein complex component-7) [23] and activated caspase-3. Paraffin-embedded fragments were sectioned (5 µm thickness) and placed on immunohistochemical slides (Dako, Santa Clara, CA, USA). Here, sections from each of the three groups (Bouin, NBF and form acetic acid) were placed on the same slide to exclude sources of technical variation and to facilitate comparison. This was carried out for each of the time points evaluated. Slides were allowed to dry overnight then were deparaffinized and rehydrated in xylene and graded concentrations of ethanol, respectively. Activated caspase-3 and Ki-67 antigens were retrieved at 97 °C for 20 min in antigen retrieval solution pH 6.0, while MCM-7 antigen was retrieved in antigen retrieval solution pH 6.0 (Dako EnVision Flex, Glostrup, Denmark). The slides were then loaded on the automatic immunohistochemical stainer, wherein slides were treated with peroxidase blocking reagent (Dako EnVision Flex, Glostrup, Denmark) before treatment with the primary antibodies against the three targets viz. activated caspase-3 (1:400; Cell signaling, Danvers, MA, USA), Ki-67 (ready to use clone MiB-1; Dako EnVision Flex) and MCM-7 antigen (1:100; Santa Cruz Biotechnology, Heidelberg, Germany). Subsequently, slides were treated with a HRP (horseradish peroxidase) cocktail specially coupled with secondary antibodies (Dako EnVision Flex, Glostrup, Denmark). DAB chromogen (Dako EnVision Flex, Glostrup, Denmark) was used for visualization of all the target antigens. Afterwards, slides were counterstained with hematoxylin (Dako EnVision Flex, Glostrup, Denmark). A section of feline tonsil was placed on each slide to serve as a positive control, while a negative control was obtained through the exclusion of the primary antibodies from the protocol. Finally, sections were cover slipped and allowed to dry overnight under a chemical cabinet before digital scanning with an automated slide scanner (NanoZoomer S60 Hamamatsu, Shizuoka, Japan).

#### Image Analysis (Immunohistochemistry)

Slides were evaluated with Qupath digital image software version 0.4.1 [24]. Diaminobenzidine (DAB) signals from the immunostained sections were measured for the 3 target proteins (Ki-67, MCM-7 and caspase-3) [12,18] using the following protocol. After opening a slide, classifying it as brightfield H-DAB and appropriately annotating it, the following command was executed: Analyse—cell detection—pixel size (0.25 µm)—classify—object classification—set cell density classification—nucleus mean DAB optical density (0.15 threshold)—analyze—add intensity features—pixel size (0.25 µm)—DAB channel color deconvolved—mean.

### 2.6. Statistical Analysis

Discrete data obtained from counting follicles were analyzed using chi-square and presented in proportions as percentages, while data from DAB intensity which were normally distributed (Jarque Bera test) were analyzed using two-way multivariate analysis of variance (MANOVA) with Tukey’s post hoc tests. A confidence interval of 95% was considered for all analyses.

## 3. Results

### 3.1. Histology

**Figure 3:**
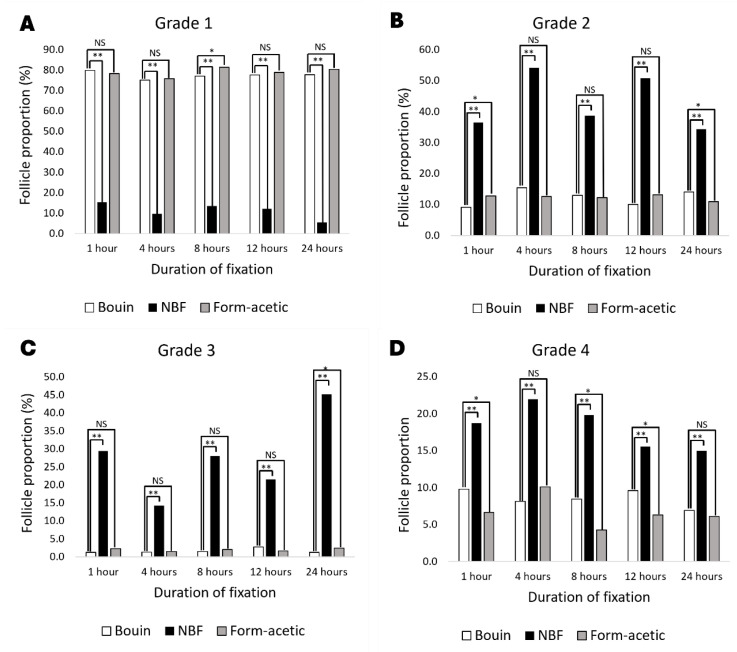
The proportion of different follicle grades in feline ovarian tissue fixed with different fixatives and fixation periods. (**A**) proportion of grade 1 (morphologically intact) follicles; (**B**) proportion of grade 2 follicles; (**C**) proportion of grade 3 follicles; (**D**) proportion of grade 4 follicles; NBF = neutral buffered formalin; * = statistically significant (*p* < 0.05); ** = extremely significant (*p* < 0.0001); NS = not statistically significant (*p* > 0.05).

**Figure 4:**
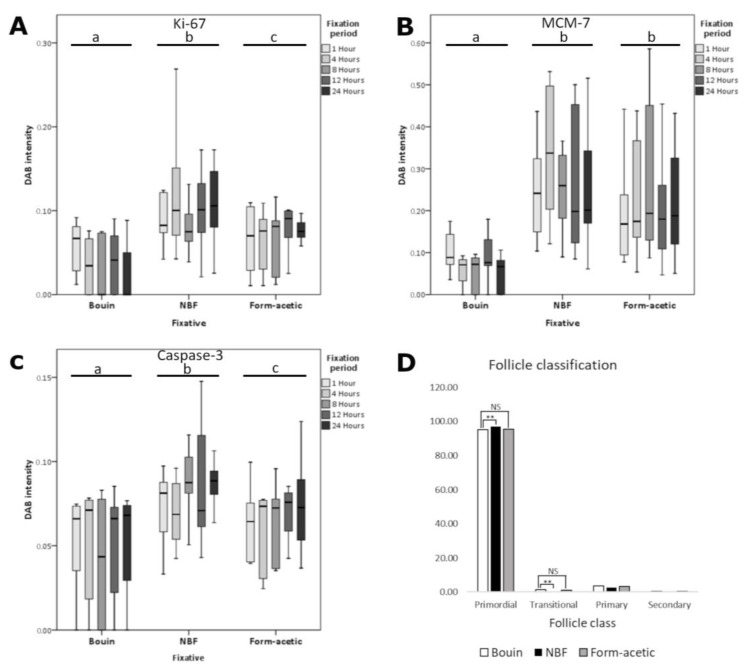
Immunohistochemical signal intensity and follicle classes in feline ovarian tissue fixed with different fixatives. (**A**) Ki-67 diaminobenzidine (DAB) intensity plot in the different groups; (**B**) MCM-7 DAB intensity distribution in the different groups; (**C**) Caspase-3 DAB intensity distribution in the different groups; (**D**) proportion (%) of different classes of follicles based on developmental stage; NBF = neutral buffered formalin. Different letters on the horizontal bars on each fixative represent statistical significance; ** = extremely significant (*p* < 0.0001); NS = not statistically significant (*p* > 0.05).

**Figure 5:**
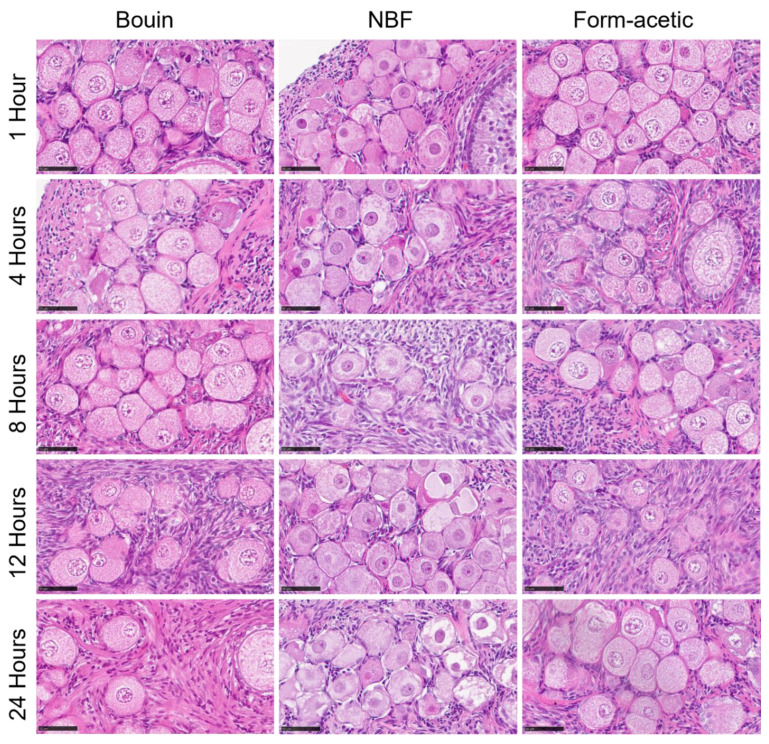
Hematoxylin- and eosin-stained micrographs of feline ovarian tissue representatives of the different fixatives and the five fixation periods. NBF = neutral buffered formalin. Scale bar = 50 µm.

The proportion of different follicle grades is presented in Figure 3. It is evident that grade 1 or morphologically intact follicles were significantly lower in NBF when compared with Bouin and form acetic acid fixatives, wherein no difference exist except at 8 h of fixation. On the other hand, NBF was higher (*p* < 0.05) in all the other grades, while Bouin and form acetic acid were lower and statistically the same except at 1 and 24 h in grade 2, at 24 h in grade 3 and at 1, 8 and 12 h in grade 4. The proportion of different classes of follicles based on the developmental stage for the five fixation periods combined is presented in Figure 4D, showing a generally high proportion of primordial follicles. The Bouin and form acetic acid fixed groups were statistically the same in all the different classes of follicles, while the NBF fixed group presented a higher (*p* < 0.05) proportion of primordial follicles with a corresponding lower (*p* < 0.05) proportion of transitional follicles. Additionally, the authors observed sectioning difficulties with NBF-fixed samples, while the opposite was observed in the case of Bouin and form acetic acid. Representative images of hematoxylin- and eosin-stained slides are presented in Figure 5.

### 3.2. Immunohistochemistry

**Figure 6:**
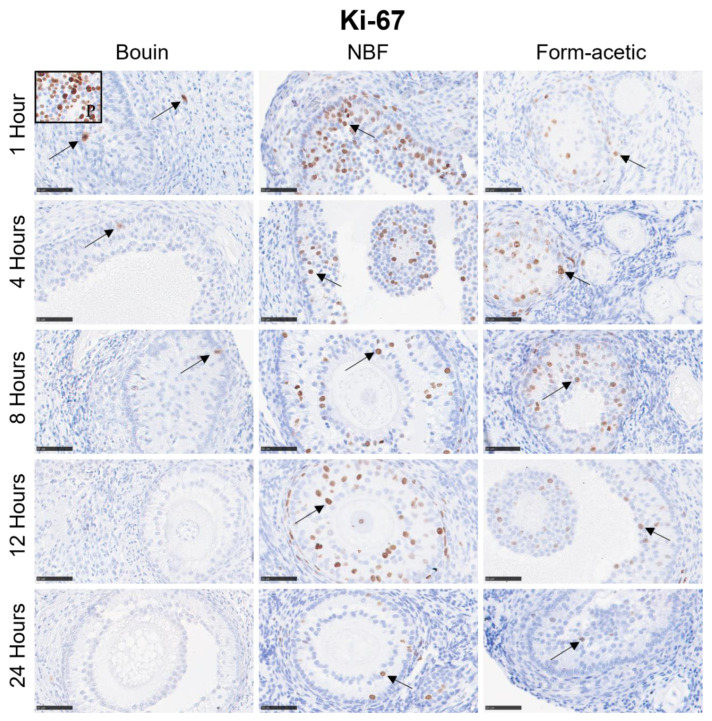
Representative immunohistochemical micrographs of feline ovarian tissue for the different fixatives and the five fixation periods immunostained with Ki-67 antibodies showing the positive brown diaminobenzidine nuclear signal (arrows) in granulosa cells. NBF = neutral buffered formalin; black framed thumbnail (P) = positive control. Scale bar = 50 µm.

**Figure 7:**
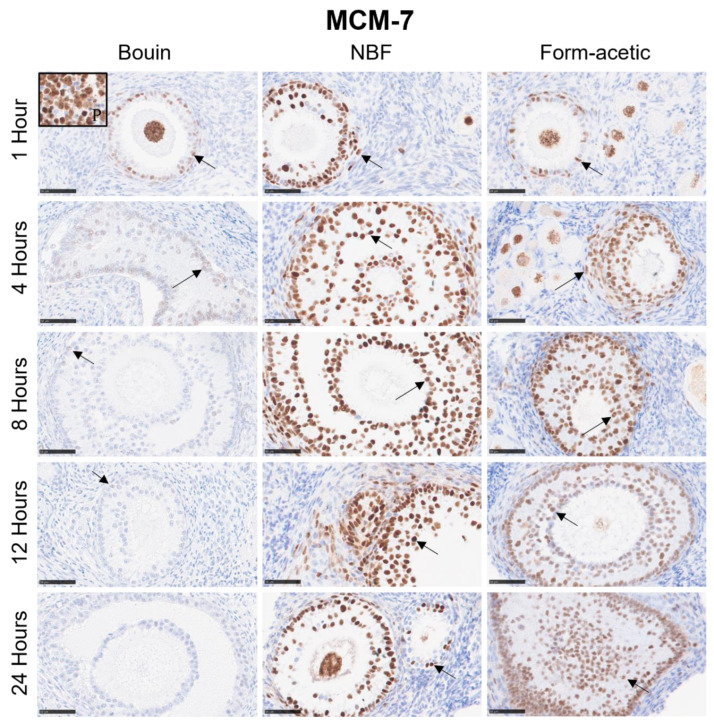
MCM-7 immunostained sections of feline ovarian tissue representative of the different fixatives and the five fixation periods showing positive brown diaminobenzidine nuclear signal (arrows) in granulosa cells. NBF = neutral buffered formalin; black framed thumbnail (P) = positive control. Scale bar = 50 µm.

**Figure 8:**
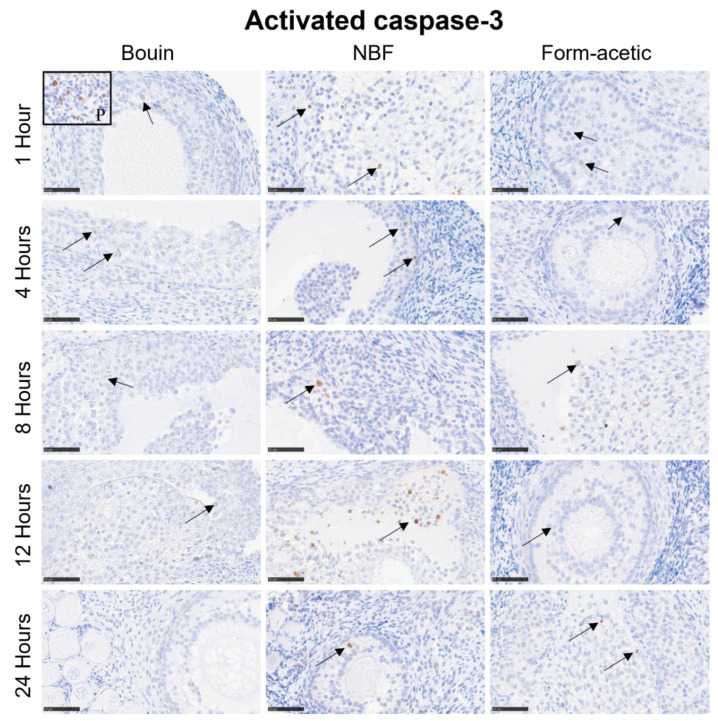
Caspase-3 immunostained sections of feline ovarian tissue representative of the different fixatives and the five fixation periods showing positive brown diaminobenzidine nuclear signal (arrows) in granulosa cells. NBF = neutral buffered formalin; black framed thumbnail (P) = positive control. Scale bar = 50 µm.

Mean DAB signal intensity data are presented as box plots in Figure 4A–C for Ki-67, MCM-7 and caspase-3 proteins, respectively. Generally, Bouin fixative had the lowest mean DAB intensity (*p* < 0.05) in all the three targets, while NBF had the highest (*p* < 0.05) in Ki-67 and caspase-3, while in MCM-7, it was no different from form acetic acid. There was no significant variation between the five fixation periods in all the three targets. Detailed representative photomicrographs are presented in Figure 6, Figure 7 and Figure 8 for Ki-67, MCM-7 and caspase-3 targets, respectively.

## 4. Discussion

This study was carried out with the aim of finding the most suitable fixative and fixation protocol for the excellent morphology and immunostaining of feline ovarian tissue. Among the three different fixatives, namely, Bouin, NBF and form acetic acid, we reported that morphologically, Bouin and form acetic acid are the best, both in terms of cellular details and their ability to identify transitional follicles regardless of the duration of fixation. This study is the first to report on the use of form acetic acid for the fixation of feline ovarian tissue. Form acetic acid is a new compound fixative containing 5% acetic acid, which is considered to be a coagulative fixative agent in NBF [20]. Acetic acid coagulates and stabilizes nuclear proteins, thus preserving nucleic acids and enhancing the morphological parameters [7].

Despite being the most widely used ideal fixative for more than a century, NBF has been associated with morphological defects ranging from those pertaining to general tissue architecture to suboptimal staining characteristics in various tissues [16,25]. Regarding ovarian tissue fixation, NBF, when used alone, has been reported to adversely affect follicle morphology [20]. Similarly, Brito et al. [9] found poor morphology when NBF was used for fixing ovarian tissue; however, the authors argued that NBF could be suitable, but only when the fixation period is 12 h. Similarly, Rahman et al. [26] concluded that NBF is associated with morphological defects in liver and brain tissues. Bouin, on the other hand, has been reported to maintain ovarian tissue morphology [20]. It is the fixative of choice when tissue fragments are very small because the yellow coloring of the tissue facilitates proper handling during histological processing and, more importantly, when excellent nuclear detail and glycogen preservation are required [27]. In addition, Bouin fixative is a formaldehyde-based compound fixative containing acetic and picric acids as supplements, making it less prone to tissue shrinkage [7,8]. Another author reported that Bouin and Carnoy fixatives provided the best comparable morphology of bovine preantral follicles, while formalin’s results were among the lowest [28]. In this report, the statistically higher proportion of transitional follicles in Bouin and form acetic acid fixatives than in NBF may indicate the latter’s lesser ability to distinguish between transitional and primordial follicles, perhaps due to the morphological aberrations associated with NBF fixation [25].

In the current report, immunohistochemical DAB signal intensity was lowest in Bouin fixative and highest in NBF. This is not surprising because several authors have reported on the limitations of using Bouin-fixed tissues for immunohistochemistry [20,27,29,30]. However, this could be dependent on the target epitope [31]. Neutral buffered formalin is considered to be the “gold standard”; thus, most immunohistochemical protocols were optimized for NBF [18]. Ananthanarayanan et al. [27] found strong MCM-2 signals in NBF-fixed tissues, while signals were completely absent in Bouin-fixed tissues. The authors modified the immunohistochemistry protocol for Bouin-fixed sections, including the addition of prolonged incubation timings and the use of different antigen retrieval techniques, but without any success. Additionally, immunohistochemical aberrations such as cytoplasmic staining, where nuclear staining is expected, produce excess background noise in all of the antigen targets evaluated, except Ki-67, AMACR and CD34, that were comparable with NBF-fixed tissues [27]. In our findings, form acetic acid was found to be related to NBF with regard to DAB signals; for instance, there was no difference in MCM-7 staining between form acetic acid and NBF. However, this is not the case with regards to Ki-67 and caspase-3, wherein DAB signals were significantly higher in NBF than in form acetic acid, while the lowest mean DAB signals were recorded in Bouin-fixed tissues. The acetic acid in form acetic acts by coagulating nucleic acid to improve morphology but does not precipitate proteins [7]; thus, it does not impede immunorecognition and is thus supplemented in some commercial alcohol-based fixatives, notably paxgene [32,33].

The current report showed that the duration of fixation is less important with respect to both the morphological and immunohistochemical signals of feline ovarian tissue fragments. In a similar report, Goldstein et al. [34], statistically, found the same immunohistochemical signals in samples fixed from 8 h to 7 days in NBF. Our findings are equally in agreement with Webster et al. [35], who evaluated prolonged fixation periods on more than 60 antigens in several domestic animals, and their results were confirmed more recently by van Seijen et al. [36]. In contrast, some authors reported otherwise, arguing that excessive cross linking may negatively affect the sensitivity of some epitopes; thus, there is reason for caution with respect to over-fixation. Moreover, most molecular techniques are usually better with a short duration of fixation, which is acceptable provided that both the morphology and other molecular techniques remain unaltered [11,37].

At the moment, there is no single known fixative that produces both good morphological traits and good results via molecular techniques in different tissues [38]; more than one fixative may be required on the same sample for different purposes. This would be challenging, especially where few tissues are available, such as when biopsies are collected from live animals. Similarly, our study showed that Bouin fixative is good for morphology but not for immunohistochemistry, while NBF is the best in terms of ovarian tissue antigenicity but not in terms of morphology. On the other hand, form acetic acid has been found to be excellent with respect to follicular morphology and good with respect to immunohistochemical signals. This will considerably simplify ovarian tissue processing, since it would eliminate the need for different fixatives for different purposes, i.e., for morphology and immunostaining. Similarly, the positive traits of form acetic acid identified with respect to the tissue sectioning process are energizing.

## 5. Conclusions

Based on our findings, we conclude that form acetic acid, in the same manner as Bouin fixative, maintains ovarian tissue architecture with excellent follicular morphology, and it also maintains a reasonable DAB signal, similar to NBF, thus providing a better alternative for feline ovarian tissue studies. These findings indicate that both the morphological and immunohistochemical evaluation of feline ovarian tissues are significantly affected by the fixatives used to preserve them. There is no doubt that more studies are needed on the effect of form acetic acid on both morphology and antigenicity, perhaps with respect to other tissues.

## Figures and Tables

**Table 1:** Follicle grading and classification.

Follicle	Description
Grade 1	Follicle is spherical in shape, evenly distributed follicular cells, intact stroma, spherical oocyte, intact nucleus and nucleolus with homogenous cytoplasm.
Grade 2	Follicle is spherical in shape, evenly distributed follicular cells, intact stroma and spherical oocyte, misshapen nucleus and/or not homogenous cytoplasm.
Grade 3	Follicular cells pulled away from the stroma but oocyte spherical.
Grade 4	Follicular cells pulled away from the stroma and/or oocyte misshapen, vacuolated, pyknotic nucleus and/or disorganized granulosa cells.
Primordial	Oocyte surrounded by flattened follicular cells.
Transitional	Some flattened follicular cells have been converted to cuboidal cells.
Primary	Oocyte surrounded by cuboidal follicular cells.
Secondary	Antral space appears.

## Data Availability

The data are contained in the manuscript; the corresponding author may be contacted for further inquiries.
